# Cyclin D1 silencing suppresses tumorigenicity, impairs DNA double strand break repair and thus radiosensitizes androgen-independent prostate cancer cells to DNA damage

**DOI:** 10.18632/oncotarget.6579

**Published:** 2015-12-12

**Authors:** Francesco Marampon, Giovanni Gravina, Xiaoming Ju, Antonella Vetuschi, Roberta Sferra, Mathew C. Casimiro, Simona Pompili, Claudio Festuccia, Alessandro Colapietro, Eugenio Gaudio, Ernesto Di Cesare, Vincenzo Tombolini, Richard G. Pestell

**Affiliations:** ^1^ University of L'Aquila, Department of Biotechnological and Applied Clinical Sciences, L'Aquila, Italy; ^2^ Department of Cancer Biology, Medical Oncology and Sidney Kimmel Cancer Center, Thomas Jefferson University, Philadelphia, Pennsylvania, USA; ^3^ Department of Human Anatomy, “La Sapienza” University of Rome, Rome, Italy; ^4^ Department of Radiotherapy, Policlinico Umberto I “Sapienza” University of Rome, Rome, Italy

**Keywords:** cyclin D1, prostate cancer, radiotherapy, DNA double-strand break repair, NHEJ

## Abstract

Patients with hormone-resistant prostate cancer (PCa) have higher biochemical failure rates following radiation therapy (RT). Cyclin D1 deregulated expression in PCa is associated with a more aggressive disease: however its role in radioresistance has not been determined. Cyclin D1 levels in the androgen-independent PC3 and 22Rv1 PCa cells were stably inhibited by infecting with cyclin D1-shRNA. Tumorigenicity and radiosensitivity were investigated using *in vitro* and *in vivo* experimental assays. Cyclin D1 silencing interfered with PCa oncogenic phenotype by inducing growth arrest in the G1 phase of cell cycle and reducing soft agar colony formation, migration, invasion *in vitro* and tumor formation and neo-angiogenesis *in vivo*. Depletion of cyclin D1 significantly radiosensitizes PCa cells by increasing the RT-induced DNA damages by affecting the NHEJ and HR pathways responsible of the DNA double-strand break repair. Following treatment of cells with RT the abundance of a biomarker of DNA damage, γ-H2AX, was dramatically increased in sh-cyclin D1 treated cells compared to shRNA control. Concordant with these observations DNA-PKcs-activation and RAD51-accumulation, part of the DNA double-strand break repair machinery, were reduced in shRNA-cyclin D1 treated cells compared to shRNA control. We further demonstrate the physical interaction between CCND1 with activated-ATM, -DNA-PKcs and RAD51 is enhanced by RT. Finally, siRNA-mediated silencing experiments indicated DNA-PKcs and RAD51 are downstream targets of CCND1-mediated PCa cells radioresistance. In summary, these observations suggest that CCND1 is a key mediator of PCa radioresistance and could represent a potential target for radioresistant hormone-resistant PCa.

## INTRODUCTION

Prostate cancer (PCa) is the most commonly diagnosed male malignancy and the second leading cause of cancer death in men. Radiation therapy (RT), considered as a major therapeutic modality for PCa treatment, is a non-invasive outpatient therapy that can be used alone, as adjuvant to surgery and/or combined with androgen deprivation therapy [1]. Although, RT generally results in an excellent initial response, some patients relapse locally and/or systemically, indicating that a resistant population of cancer cells may have survived the RT [1–2]. Clinical observation shows that patients with androgen-independent PCa appear to have higher biochemical failure rates after RT. Previous studies also indicate that the response to RT is different between androgen-dependent and androgen-independent PCa cells, indicating that molecular events mediated by androgen may also function in radiosensitization and that androgen-independency may be associated with radiation resistance in PCa [3]. Although various genetic, epigenetic and molecular abnormalities have been associated with radiation resistance in PCa [4], the molecular mechanisms responsible of radiation resistance and relationship with androgen-independent PCa phenotype remains unknown. Understanding these phenomena could lead to new molecular targets and more directed therapy able to improve the RT efficiency.

RT promotes cytotoxicity by inducing several forms of DNA damage such as double-strand breaks (DSBs). Two mechanisms exist to repair mammalian DSBs: non-homologous end joining (NHEJ) and homologous recombination (HR) that are chosen depending in part upon the phase of the cell cycle and chromatin context [5]. It is generally considered that accurate repair by HR is restricted to S and G2 phases of the cell cycle, whereas NHEJ is predominant in G_0_/G_1_ cells [5–6]. Ataxia telangiectasia mutated (ATM) and the DNA dependent protein kinase catalytic subunit (DNA-PKcs) play a key roles in the DSBs response, via HR and NHEJ, respectively. Once activated, ATM and DNA-PKcs regulate a wide spectrum of downstream targets that are involved in the DNA damage repair process, cell cycle regulation and apoptosis [7]. Tumor cells escape from RT induced cytotoxicity by activating a complex network of pathways able to remove DSBs and to permit cell cycle progression [5]. Furthermore, RT can also simultaneously induce multiple pro-survival signaling pathways which can lead to suppression of apoptosis, induction of cell cycle arrest and/or initiation of DNA repair. These signaling pathways act in concert to reduce the magnitude of radiation-induced cytotoxicity and promote the development of radioresistance in cancer cells [8–9]. The identification of the molecular mechanisms involved in the DNA damage response raises the possibility to specifically target cancer cells inducing a radiosensitization [10–11].

The D-type cyclin family, composed of three proteins, Cyclin D1, D2 and D3, regulates the G1/S-phase transition of proliferating cells [12]. Of the three D-type cyclins, it is cyclin D1 overexpression that is predominantly associated with human tumorigenesis and cellular metastases. Amplification or overexpression of cyclin D1 plays pivotal roles in the development of a subset of human cancers including parathyroid adenoma, breast cancer, colon cancer, lymphoma, melanoma, and prostate cancer [13–16]. Increasing evidences show that cyclin D1 governs DNA damage repair through forming and regulating several multi-protein DNA repair complexes that participate in DNA repair [17–22]. Despite the evidence collected implicating cyclin D1 in DNA repair and radioresistance of cancer cells, little is known about its role in prostate cancer cells and the relationship between cyclin D1, androgen independency and radioresistance.

In this manuscript, we investigated whether silencing cyclin D1 affects the radiosensitivity of androgen-independent, androgen-receptor negative PC3 [23–24] and androgen-independent, androgen-receptor positive 22Rv1 cells [25] by deploying *in vitro* and *in vivo* models systems. We generated stably infected cell lines expressing shRNA against cyclin D1. Herein, cyclin D1 depletion suppressed the tumorigenic phenotype and increased the radiosensitivity of PCa cell lines both *in vitro* and *in vivo*. Cyclin D1 silencing impaired the DSBs repair mediated by the NHEJ and HR molecular machinery. These results substantiate the idea that cyclin D1 is an important regulator of tumorigenesis and radioresistance of androgen-independent PCa cells.

## RESULTS

### Silencing cyclin D1 affects the cell lines PC3 and 22Rv1 oncophenotype

**Figure 1:**
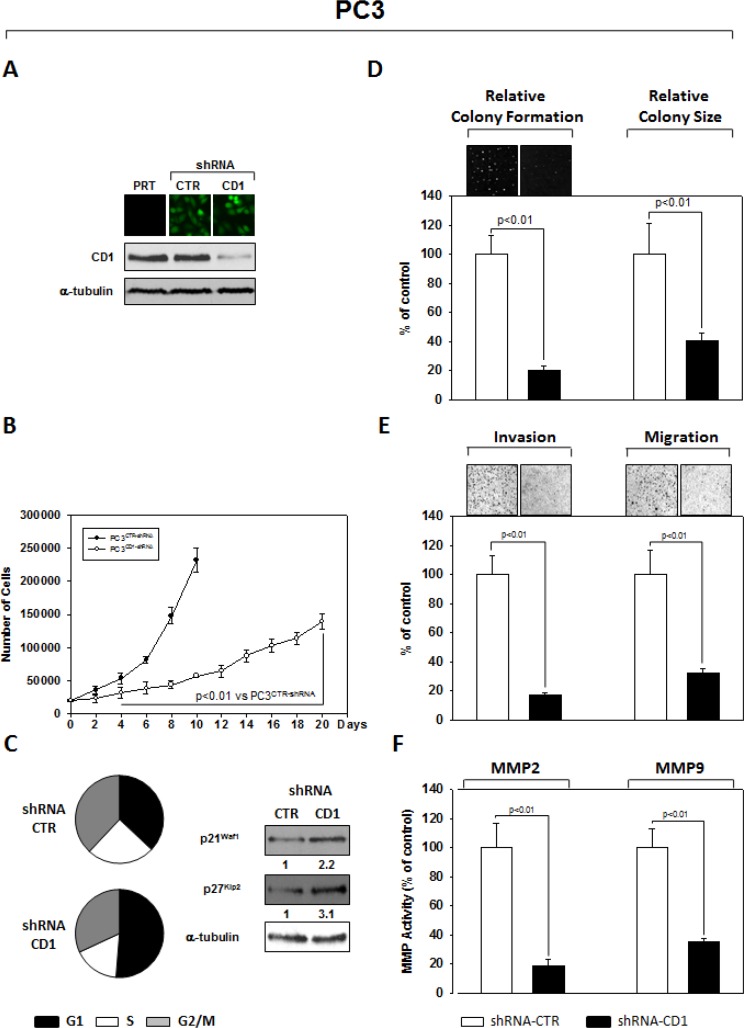
Stable and Specific Silencing of cyclin D1 inhibits PC3 onco-phenotype (**A**) Parental PC3 (PRT), GFP-positive PC3 cells, stably infected with shRNA-cyclin D1 (CD1) vs. shRNA-control (CTR) sequence (CTR), were selected by FACS sorting and examined for cyclin D1 protein expression by immunoblotting. (**B**) Cell growth assay, (**C**) cell cycle distribution by FACS and p21^waf1^, p27^KIP2^ by immunoblotting, (**D**) soft agar assay and relative colony size, (**E**) invasion- and migration-assay and (**F**) the activation status of MMP-2 and MMP-9 by ELISA assay were performed. The data presented in Figure 1B, 1C, 1D, 1E and 1F represent the mean ± SD of 3 independent experiments. Statistical analysis was performed using the Student's *t*-test, *P* < 0.01. For immunoblotting, α-tubulin expression shows equal loading. Similar results were obtained in *n* = 3 experiments.

**Figure 2:**
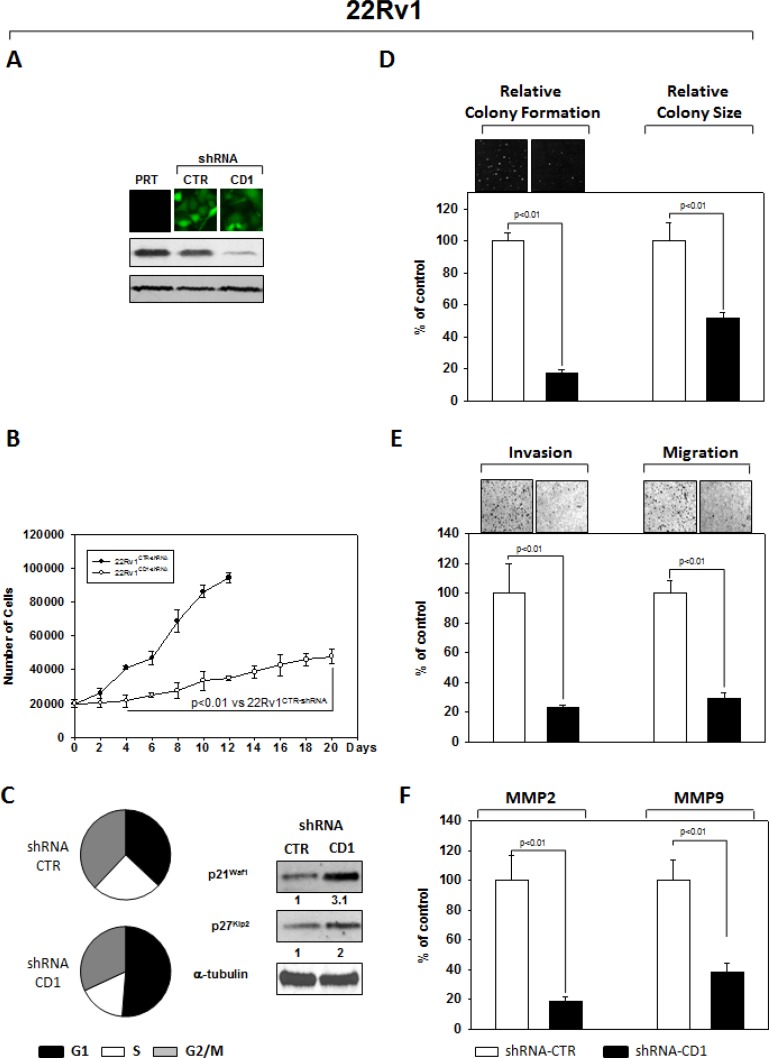
Stable and Specific Silencing of cyclin D1 inhibits 22Rv1 onco-phenotype (**A**) Parental 22Rv1 (PRT), GFP-positive 22Rv1 cells, stably infected with shRNA-cyclin D1 (CD1) vs. shRNA-control (CTR) sequence (CTR), were selected by FACS sorting and examined for cyclin D1 protein expression by immunoblotting. (**B**) Cell growth assay, (**C**) cell cycle distribution by FACS and p21^waf1^, p27^KIP2^ by immunoblotting, (**D**) soft agar assay and relative colony size, (**E**) invasion- and migration-assay and (**F**) the activation status of MMP-2 and MMP-9 by ELISA assay were performed. The data presented in Figure 1B, 1C, 1D, 1E and 1F represent the mean ± SD of 3 independent experiments. Statistical analysis was performed using the Student's *t*-test, *P* < 0.01. For immunoblotting, α-tubulin expression shows equal loading. Similar results were obtained in *n* = 3 experiments.

**Figure 3:**
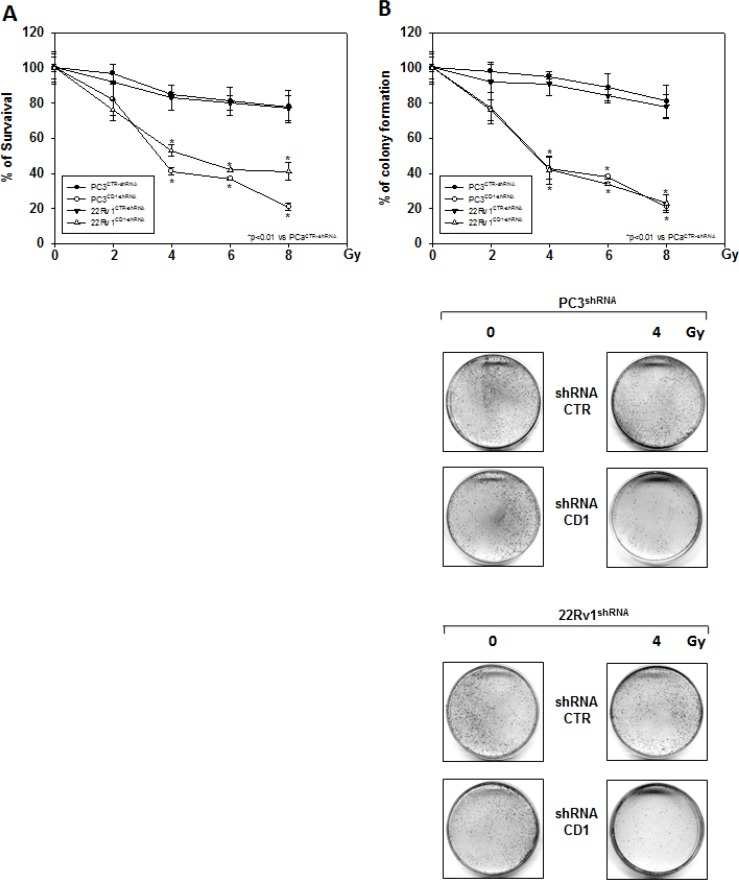
Silencing cyclin D1 radiosensitizes PC3 and 22Rv1 cell lines *in vitro* shRNA-cyclin D1- (CD1) and shRNA-control-transduced (CTR) PC3 and 22Rv1 cells were irradiated with various doses (0–8 Gy): (**A**) MTT and (**B**) (Upper Panel) colony formation assays were performed. The data presented in Figure 3A and 3B Upper Panel represent the mean ± SD of 3 independent experiments. Statistical analysis was performed using the Student's *t*-test, *P* < 0.01. Figure 3B Lower Panel shows the effects of 4 Gy irradiation on shRNA-cyclin D1- and shRNA-control-transduced PC3 and 22Rv1 cells.

An shRNA sequence versus cyclin D1, cloned in the GFP-expressing pLVTHM plasmid, was used to knock down expression of *cyclin D1* in the PC3 (Figure 1) and 22Rv1 (Figure 2) PCa cell lines. As extensively described in the Materials and Methods section, GFP-positive cells, isolated by FACS sorting for GFP+ cells, were expanded and examined by western blot for the cyclin D1 protein abundance. PC3- (Figure 1A) and 22Rv1- (Figure 2A) shRNA-cyclin D1 infected cells showed a significant reduction in cyclin D1 protein expression (Figures 1A PC3 and 2A 22Rv1). We conducted experiments that compared tumorigenicity of the shRNA-cyclin D1- versus shRNA-control-transduced cells. Delay in cell growth, cell cycle analysis, soft agar colony formation-, migration- and invasion-abilities were investigated. Silencing cyclin D1 leds to a delay in growth of PCa cells: PC3- and 22Rv1-Cyclin D1-shRNA transduced cells respectively demonstrated a 5-fold decrease at 10-days and a 2-fold decrease at 12 days in proliferation compared to control-shRNA transduced cells (Figures 2B and 3B). FACS analysis shows that silencing cyclin D1 increased the proportion of PC3 (Figure 1C) and 22Rv1 (Figure 2C) cells in G_1_ phase and up-regulated the p21^Waf1^ and p27^Kip2^ cell cycle inhibitor protein expression levels. Figure 1D (PC3) and 2D (22Rv1) show that silencing cyclin D1 reduced by 80% (PC3) and 82.5% (22Rv1) the ability to form colony in soft agar and by 69% (PC3) and 48% (22Rv1) the colony medium size. Figure 1E (PC3) and 2E (22Rv1) show that cyclin D1 silencing reduced by 83% (PC3) and 77% (22Rv1) invasion and by 68% (PC3) and 71% (22Rv1) migration abilities. Given the observed effects on invasion and migration, the matrix metallopeptidase 2 and 9 (MMP-2 and -9) activities were assessed by ELISA assay. Figures 1F and 2F show that cyclin D1 depletion reduced the MMP-2 activity by 81% (PC3) and 82% (22Rv1), the MMP-9 activity by 65% (PC3) and 62% (22Rv1).

### Cyclin D1 governs the radioresistant phenotype of PC3 and 22Rv1 cell lines *in vitro* and *in vivo*

**Figure 4:**
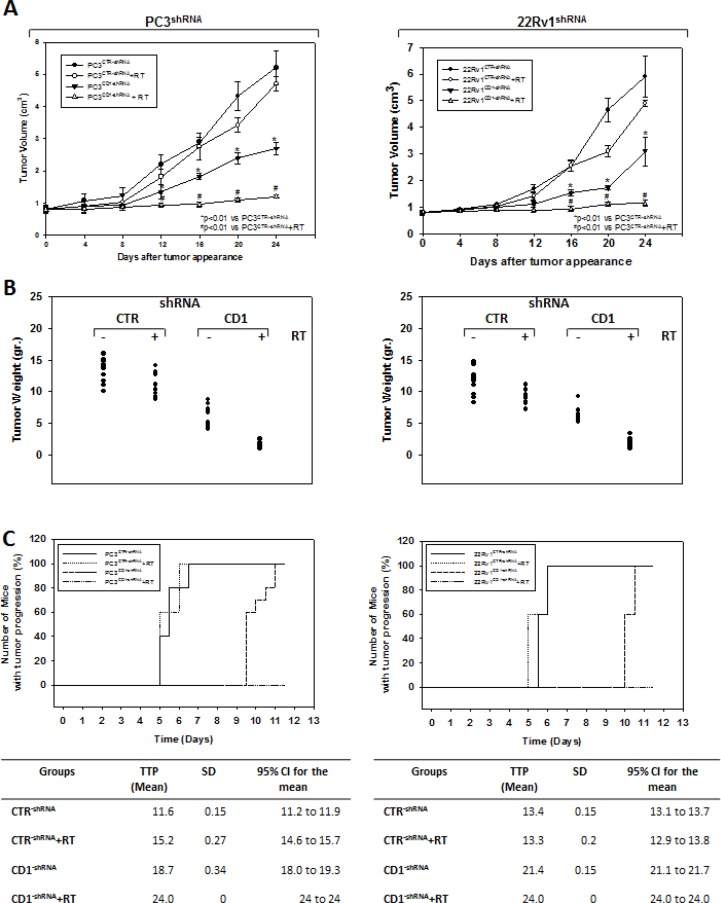
Silencing cyclin D1 radiosensitizersPC3 and 22Rv1 cell lines *in vivo* Mice xenografted with shRNA-cyclin D1- (CD1) and shRNA-control-transduced (CTR) PC3 (Left Panel) or 22Rv1 (Right Panel) cells subjected to radiation treatment (5 fractions of 2 Gy were delivered over 5 consecutive days for a total dose of 10 Gy) starting when the tumor volume reached 0.5–1.0 mm^3^ (T0). (**A**) Tumor volume, (**B**) tumor weights and (**C**) number of mice with tumor progression.

**Figure 5:**
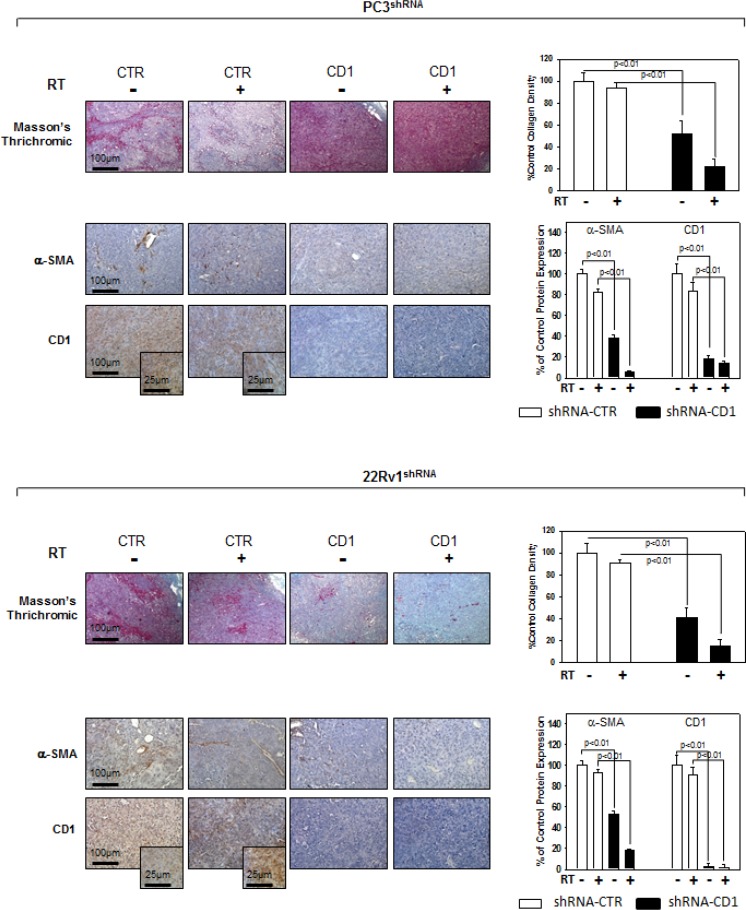
Immunohistochemistry, α-SMA and cyclin D1 staining in sections from mice xenografted with PC3 and 22Rv1 cell lines and subjected to radiotherapy Mice xenografted with shRNA-cyclin D1- (CD1) and shRNA-control-transduced (CTR) PC3 (Upper Panel) or 22Rv1 (Lower Panel) cells subjected to radiation treatment (5 fractions of 2 Gy were delivered over 5 consecutive days for a total dose of 10 Gy) starting when the tumor volume reached 0.5–1.0 mm^3^. Masson's thrichromic staining, α-SMA and cyclin D1 staining. Original Magnification 10X. Insert: original magnification 40X. (A and B Right Panel) Protein quantification. The data presented represent the mean ± SD of 3 independent experiments. Statistical analysis was performed using the Student's *t*-test, *P* < 0.01.

We investigated the effect of silencing cyclin D1 combined with radiotherapy of human PCa cell lines *in vitro* and *in vivo*. For the *in vitro* experiments, control- and cyclin D1-shRNA transduced cells were treated with several doses (0–8 Gy) of radiation. MTT assay (Figure 3A), performed after 24 hrs post irradiation, shows that silencing cyclin D1 significantly reduced the PC3 and 22Rv1 cells survival. Colony formation assay was performed to determine cell reproductive death after treatment with ionizing radiation. Concordant with the delay in growth intrinsic to cyclin D1-shRNA transduced cells, colonies from control-shRNA transduced cells could be counted after 14 days while those from cyclin D1-shRNA transduced cells could not be evaluated until 45 days post irradiation. As shown in Figure 3B, a significant reduction in number of cell colonies were observed in cyclin D1-shRNA + RT groups compared to control-shRNA group at all tested doses of radiation in both cell lines. For *in vivo* experiments, RT treatment (5 fractions of 2 Gy delivered over 5 consecutive days for a total dose of 10 Gy) was started when tumor volume reached 0.5–1.0 mm^3^ (T0). Tumor volumes were measured every 4 days for a period of 24 days (Figure 4A) while tumor weight was measured at the end of the experiment (Figure 4B). PC3- and 22Rv1-cyclin D1-shRNA xenografted mice grew significantly less with respect to control groups and RT treatment decreased growth further (Figure 4A). These effects were confirmed by measuring tumor weight (Figure 4B). The number of mice with tumor progression significantly differed across the groups and this was confirmed by the mean values of TTP (Figure 4C). In the mice xenografted with tumor cells expressing cyclin D1-shRNA, tumor progressions occurred within 12 and 14 days after the T0 in PC3 and 22Rv1 tumor cells, respectively (Figure 4C). The mean TTP of these xenografts was of 11.6 days (95% CI 11.3 to 11.9) in PC3 and 13.4. days (95% CI 13.1 to 13.7) in 22Rv1, respectively. Although an effect of RT, in terms of tumor weight and tumor volume, was observed in 22Rv1 expressing cyclin D1, a negligible improvement in the TTP was documented in this xenograft model [13.0. days (95% CI 12.9 to 13.8)] with respect to controls. In contrast PC3 cells expressing cyclin D1 [15.2. days (95% CI 14.7 to 15.7)] demonstrated, an improvement of the RT response compared to controls. Interestingly, when cyclin D1 was silenced the TTP was significantly prolonged (*p* < 0.05) with respect to controls and RT resulted in no tumor progression in both xenograft models (Figure 4C). Masson trichromic staining was performed to evaluate collagen deposition and changes in the prostatic parenchymal architecture (Figure 5, Masson Trichromic). Tumor masses from control-shRNA transduced cells showed the presence of nodules surrounded by bundles of connective tissue together with a massive neovascularization close to the nodules and throughout the entire parenchyma; RT treatment did not induce any substantial modification. Silencing cyclin D1 induced a marked changes in the morphological pattern with an almost normal parenchymal architecture, a normal collagen distribution; these effects were enhanced by RT treatment. Collagen density was also evaluated by colorimetric assay (Figure 5, % Control Collagen Density). Silencing cyclin D1 *per se* decreased collagen density by 48.2% (PC3) and 59.1% (22Rv1): these effects were potentiated by RT treatment resulting in a further decrease of the collagen density by 78.3% (PC3) and 85.3% (22Rv1). RT did not modify collagen density of control-shRNA transduced cells. Vascular response to silencing cyclin D1 was analyzed by α-SMA staining and protein expression quantification (Figure 5, α-SMA). There was significant difference in α-SMA protein expression. Compared to control-shRNA-transduced group, in cyclin D1-shRNA transduced group the α-SMA protein expression was reduced by 61.5% (PC3) and 47.2% (22Rv1). These effects were enhanced by RT treatment resulting in a further α-SMA protein expression reduction by 94.2% (PC3) and 81.3% (22Rv1). Figure 5A and 5B (cyclin D1) depicts the immunohistochemical analysis and protein expression quantification for cyclin D1.

### Silencing cyclin D1 radiosensitizes PCa cells by impairing the NHEJ-DNA-PKcs and HR-ATM-RAD51 pathways responsible for the DNA double-strand break repair

**Figure 6:**
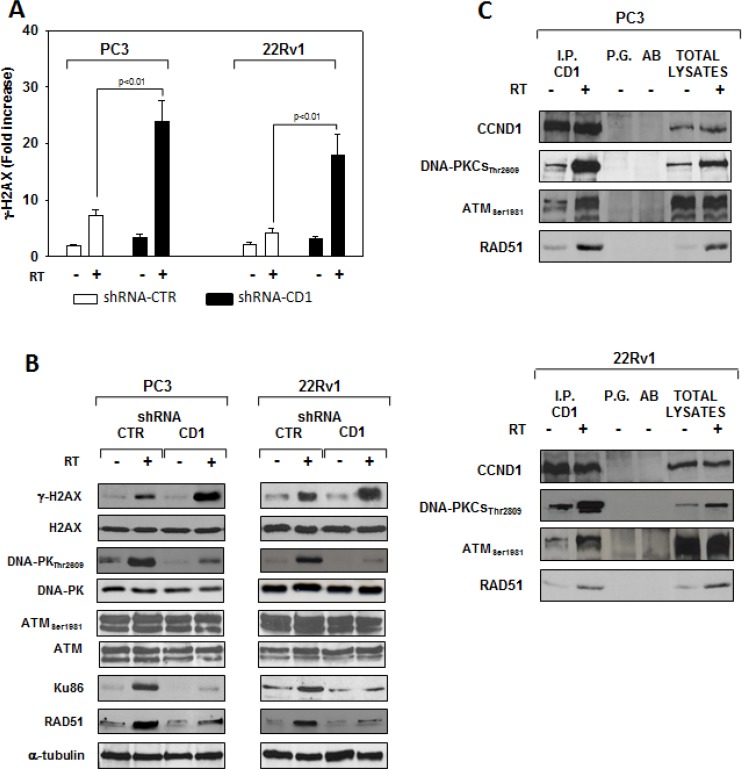
cyclin D1 depletion do not radiosentize via inducing apopotosis but rather by impairing the molecular machinery responsible of the DNA double-strand break repair shRNA-cyclin D1- (CD1) and shRNA-control-transduced (CTR) PC3 or 22Rv1 cells irradiated with a single dose of 4 Gy. (**A**) 6 hours post RT, H2AX activation status was investigated using and ELISA assay for γ-H2AX; the data represent the mean ± SD of 3 independent experiments. Statistical analysis was performed using the Student's *t*-test, *P* < 0.01. (**B**) Cell lysate were processed for the indicated proteins by immunoblotting; α-tubulin demonstrates equal loading. Similar results were obtained in *n* = 3 experiments. (**C**) Cyclin D1-DNA-PKCs, -ATM, -RAD51 heterodimers in PC3 (Upper Panel) or 22Rv1 (Lower Panel) cells untreated (−) or treated (+) with RT (4 Gy). 6 hours post RT, cyclin D1 was immunoprecipitated (IP) with an anti-cyclin D1 polyclonal antibody from extracts containing equal amounts of total proteins and subsequently analyzed by immunoblotting with a cyclin D1, DNA-PKCs_Thr2809_, ATM_Ser1981_ or RAD51 monoclonal antibodies. Similar results were obtained in *n* = 2 experiments.

**Figure 7:**
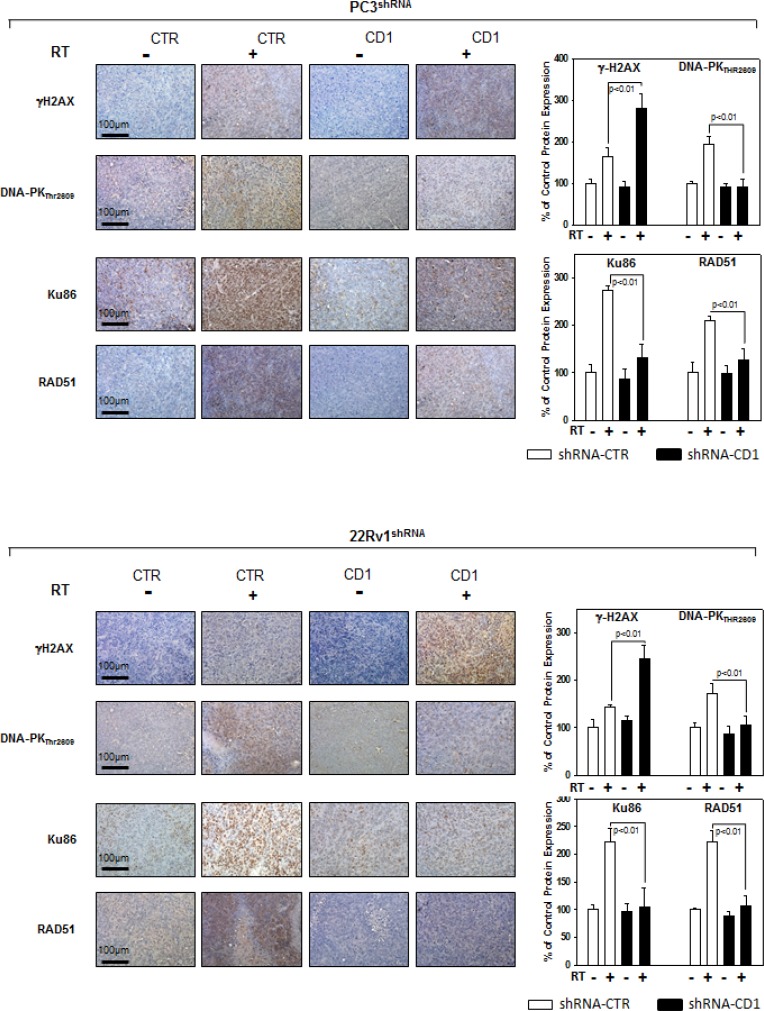
Cyclin D1staining in sections from mice xenografted with PC3 and 22Rv1 cell lines and subjected to radiotherapy Mice xenografted with shRNA-cyclin D1- (CD1) and shRNA-control-transduced (CTR) PC3 (Upper Panel) or 22Rv1 (Lower Panel) cells subjected to radiation treatment (5 fractions of 2 Gy were delivered over 5 consecutive days for a total dose of 10 Gy) starting when the tumor volume reached 0.5–1.0 mm^3^: γ-H2AX, Pospho-DNA-PKCs, Ku86 and RAD51 staining and protein quantification. The data represent the mean ± SD of 3 independent experiments. Statistical analysis was performed using the Student's *t*-test, *P* < 0.01.

**Figure 8:**
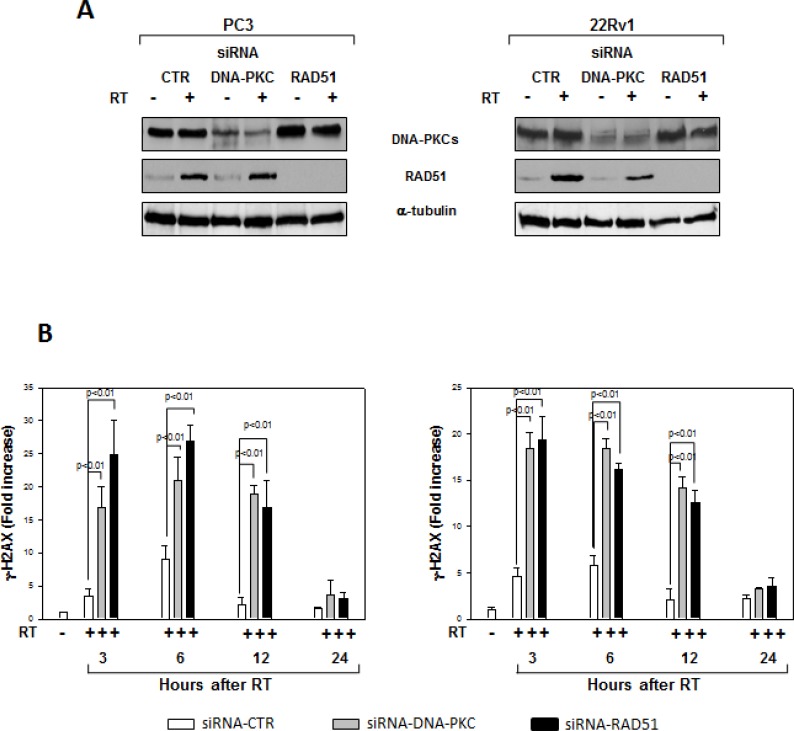
Effects of RAD51 or DNA-PKcs siRNA on DNA damage (**A**) Reduced RAD51 or DNA-PKcs expression by RAD51 or DNA/PKcs siRNA. Western blot analysis performed using lysates isolated from PC3 (Left Panel) or 22Rv1 (Right Panel) cells expressing RAD51, DNA-PKcs or Control siRNA and subjected to 4 Gy of RT. Cell lysates were processed for the indicated proteins by immunoblotting; α-tubulin expression shows equal loading. (**B**) H2AX activation status was investigated by using an ELISA assay for γ-H2AX at different time from RT treatment; the data represent the mean ± SD of 3 independent experiments. Statistical analysis was performed using the Student's *t*-test, *P* < 0.01. Similar results were obtained in *n* = 3 experiments.

We assessed whether silencing cyclin D1 may sensitize PCa cells to ionizing radiation by inducing apoptosis and/or promoting the DNA damage and impairing the molecular mechanisms of DSBs repair. To this purpose, control- and cyclin D1-shRNA transduced cells were irradiated with a single dose of 4 Gy and cell lysates were processed 6 hours after RT. The analysis of apoptotic markers show that 4 Gy RT treatment did not induce significant apoptosis of cyclin D1-versus control-shRNA transduced cells (Data not shown). We next tested the abundance of γ-H2AX levels, a biomarker for DNA double-strand breaks, as well as the activation status and/or abundance of ATM, DNA-PKcs, RAD51 and RAD86 that govern the DNA-DSBs repair machinery. ELISA (Figure 6A) and western blot (Figure 6B, γ-H2AX) showed that in the presence of silencing cyclin D1, RT significantly increased the DNA damage as suggested by the upregulation of γ-H2AX protein expression levels. Cyclin D1 depletion counteracted DNA-PKcs_Thr2609_ phosphorylation/activation and the accumulation of RAD51 and Ku86 proteins induced by RT. No effects on ATM_Ser1981_ phosphorylation status was observed (Figure 6B). The ability of cyclin D1 to directly interact with the proteins of DNA-DSBs repair machinery was investigated. PC3 and 22Rv1 cells were irradiated with a single dose of 4 Gy and, after 6 hours, cell lysates were immunoprecipitated for cyclin D1: PKcs_Thr2609_, -ATM_Ser1981_ and RAD51 association was tested by immublotting. Cyclin D1 bound DNA-PKcs_Thr2609_, -ATM_Ser1981_ and RAD51 and the amount of activated-DNA/PK,-ATM and RAD51 was significantly increased by RT treatment (Figure 6C). The relationship between cyclin D1 and DNA-DSBs repair machinery was also tested in *in vivo* experiment on mice xenografted with control- or cyclin D1-shRNA transduced PC3 or 22Rv1 cells, treated or not with RT as already described and sacrificed 24 hours after the last RT dose (Figure 7). Also in *in vivo*, silencing cyclin D1 favored the DNA-DSBs damage, impairing the DNA-damaged repair. In presence of cyclin D1 silencing, RT increased γ-H2AX expression by 281% (PC3) and 246% (22Rv1) (Figure 7 γ-H2AX) while poor DNA-PKcs activation (DNA-PKcs_Thr2609_), Ku86 and RAD51 protein expression (Figure 7) were identified. Lastly we investigated if DNA-PKcs- and/or ATM-RAD51-pathways were downstream targets of cyclin D1-mediated radioresistance. To this end, PC3 and 22Rv1 cells subjected to DNA-PKcs or RAD51 protein silencing with specific siRNA, were irradiated with a single dose of 4 Gy: γ-H2AX levels were tested by ELISA at different time post RT treatment. siRNA transfection resulted in a downregulation of DNA-PKcs or RAD51 expression with respect to the siRNA-control-transfected cells (Figure 8A). ELISA (Figure 8B) show that DNA-PKcs or RAD51 silencing significantly favored the DNA damage induced by RT. Contrary to siRNA-control-transfected cells, in which the γ-H2AX upregulation was transient and disappeared within 12 hours, DNA-PKcs or RAD51 silencing favored a greater and more lasting effect on DNA damage, suggesting DNA-PKcs- and ATM/RAD51-pathway are downstream targets of cyclin D1 induced PCa cell radioresistance.

**Figure:**
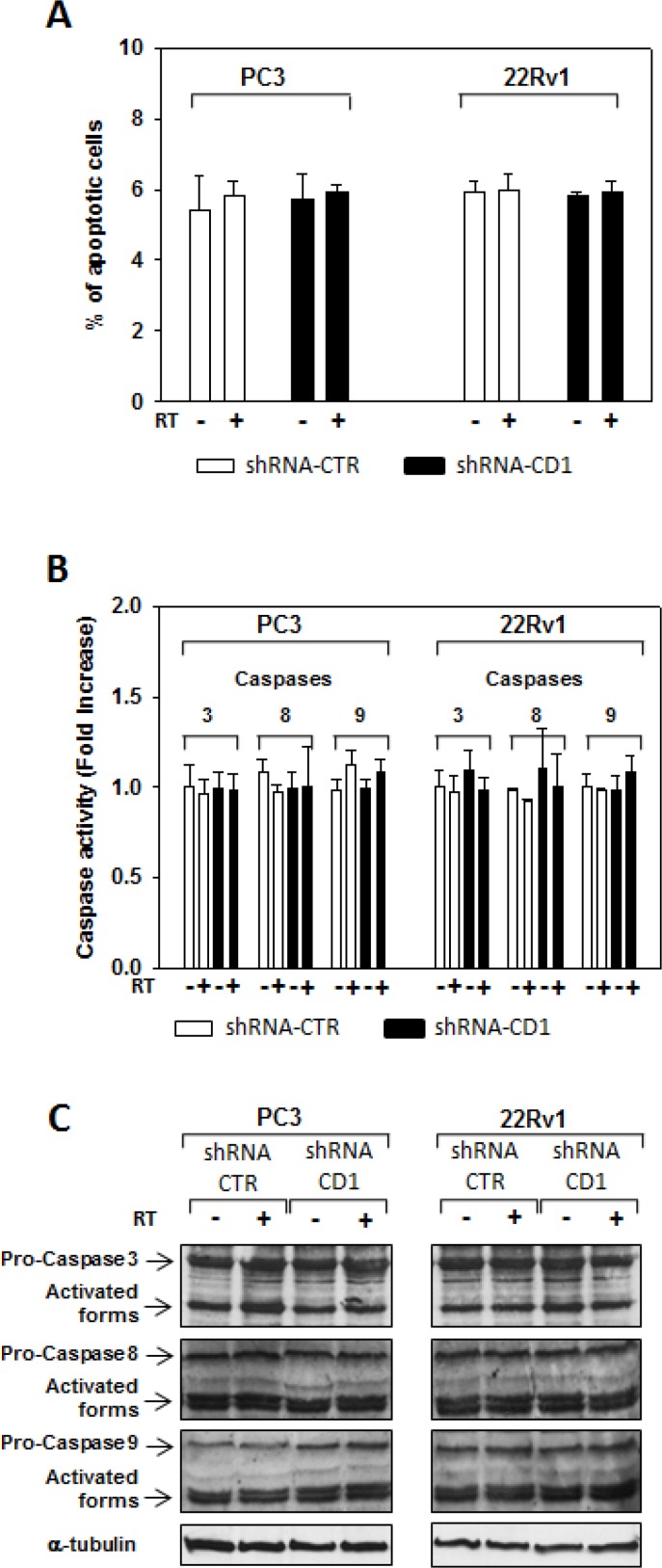
Data not shown. Cyclin D1 silencing did not radiosensitize by inducing apoptosis control- (CTR) and cyclin D1-shRNA (CD1) transduced PC3 and 22Rv1 cells were subjected to 4 Gy of irradiation. (**A**) TUNEL assay and the evaluation of caspase-3, -8 and -9 activation by ELISA (**B**) and western blott (**C**) assays were performed after 6 hours from irradiation. The data presented in A and B represent the mean ± SD of 3 independent experiments. Statistical analysis was performed using the Student's *t*-test, *P* < 0.01.

## DISCUSSION

Radiation therapy (RT) is considered the first line treatment for prostate cancer (PCa). Despite technical improvements, many patients relapse locally and/or with systemic disease, indicating the presence of a radio resistant cancer cell population [1–2]. Furthermore, several evidence show that PCa cells with an androgen-independent phenotype have higher biochemical failure rates after RT suggesting that androgen-independency may be associated with radiation resistance in PCa [3]. Cancer cells are frequently characterize by an elevated DNA repair capacity that leads to radiation resistance and targeting the DNA repair machinery could enhance the efficacy of RT [10–11]. In previous studies *Ccnd1* gene deletion was associated with reduced proliferation of prostate epithelial cells and induction of a cyclin D1 mediated gene signature that predicted poor outcome and recurrence free survival in prostate cancer patients [16]. Herein, we investigated if cyclin D1 contributes to androgen-independent PCa cells radioresistance. The role of cyclin D1 in androgen-independent PCa cells response to RT was previously unknown. By using androgen-independent, androgen-receptor negative PC3 [23–24] and androgen-receptor positive 22Rv1 [25] PCa cell lines stable infected with shRNA for cyclin D1, we show that cyclin D1 is a key regulator in controlling the DNA double strand break (DSB) repair mechanisms mediated by the non homologous end joining (NHEJ) pathway and that the silencing cyclin D1 radiosensitizes PCa cells.

D-type cyclins (D1, D2, and D3) are G1-specific cyclins that promote restriction point progression during G1 phase [12]. Amplification of individual cyclin D genes and overexpression of their encoded proteins were documented in a large proportion of human cancers [12–16]. Cyclin D1 protein expression is induced by growth factors in human PCa cell lines and is increased in a subset of PCa samples, promoting PCa cell growth [15, 30]. Previous studies show that silencing cyclin D1 reduced growth *in vitro* and *in vivo* [14, 16] while its overexpression increased cell growth and tumorigenicity of androgen-dependent, androgen receptor-expressing LnCAP cells [31]. In the current studies, silencing cyclin D1 affected the tumorigenic potential of androgen-independent PC3 and 22Rv1PCa cell lines *in vitro* and *in vivo* independently of the expression of androgen receptor. Silencing cyclin D1 in PCa cells showed a significant growth delay both *in vitro* and *in vivo*, with many cells arrested in the G1 phase of the cell cycle. Furthermore, cells cannot expand for more than 6 passages. Cancer cell tumorigenicity is characterized by the strong ability of cancer cells to invade and migrate [32]. Cyclin D1 plays an important role in cell migration [33] promoting the migratory and invasive capacity of macrophages [34], fibroblasts [35], breast epithelial cells [36] and human glioblastoma cells [37–38]. The mechanism, rather complex, is via RhoA and binding to the cytoplasmic proteins Filamin A [39], Pacsin 2 [40] and the regulation of microRNAs such as microRNA-17/20 [41]. Furthermore, cyclin D1 regulates metalloproteinase (MMPs) [42–43], traditionallyassociated with matrix remodeling, cancer invasion and with angiogenesis. Here, in characterizing the tumorigenic phenotype of cyclin D1 silenced PCa cell lines, we showed that migration and invasion abilities as well as MMP2 and MMP9 activities were drastically reduced in the absence of cyclin D1. Cancer progression, invasion, and metastasis are processes strictly correlated with angiogenesis that plays a key role in PCa disease progression [44]. Herein silencing cyclin D1 induced *in vivo* changes in tumor vascularity and structural organization, impairing the amplitude and architecture of the vascular bed. These effects could are in accordance with studies demonstrating cyclin D1depletion inhibits VEGF -stimulated growth of vascular endothelial cells causing several abnormalities to the normal organization of the vascular bed [45–46]. Our evidences indicated that cyclin D1 is essential for the maintenance of PCa cells tumorigenic abilities and that cyclin D1 depletion alone can reverts the oncogenic phenotype.

**Figure 9:**
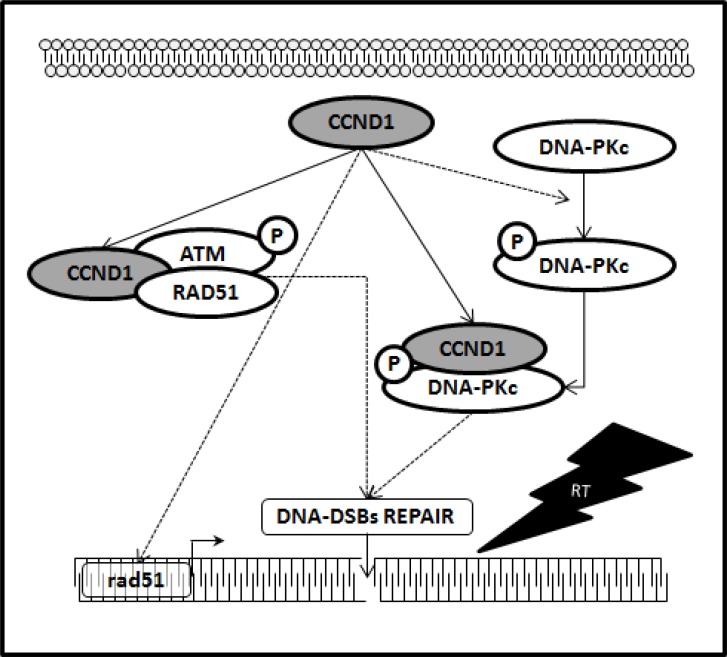
Cyclin D1 promotes NHJE and HR pathways responsible of DNA-DSBs repair Schematic representation depicting the collaboration of cyclin D1 with the molecular pathways responsible for the DNA-DSBs repair. In dashed line, the molecular mechanism by which it is necessary to verify whether the action of cyclin D1 is direct or mediated by other factors.

RT works through damaging the DNA of exposed tumor tissue leading to cell death: DNA double-strand breaks (DSBs), the most deleterious lesions, are repaired by two main pathways namely non-homologous end joining (NHEJ) and homologous recombination (HR). Activation of DSBs repair genes is one of the reasons for chemo- and radioresistance, therefore, targeting DSBs repair is an attractive strategy to eliminate cancer [6–9]. The relationship between cyclin D1 and DSBs repair machinery is largely unknown and the fact that cyclin D1 has the hallmarks of a cellular proto-oncogene suggests its possible key role in promoting DNA repair and consequently promote radioresistance of PCa cells. Previous studies reported a correlation between cyclin D1 overexpression, perturbation of the DNA repair machinery and acquisition of a radioresistant phenotype in cancer cells [17–22]. No evidences has yet collected on the relationship between cyclin D1 and radiosensitivity of androgen-independent PCa cells radioresistance. Herein, silencing cyclin D1 radiosensitizedandrogen-independent PCa cell lines both *in vitro*, reducing cells clonogenic survival, and *in vivo* impairing xenograft growth of the PCa cells after RT treatment. Cancer cells escape from RT by repairing the DNA lesions trough the activation of highly conserved enzymatic pathways: only the accumulation off rank unrepaired breaks may generate chromosomal aberrations that, after a variable number of cell cycles, induce cell death. This mode of cell death is considered the major mechanism by which solid tumors respond to clinical radiotherapy. Apoptosis is an alternative mode of cell death after RT, but appears to be preferentially expressed in embryonal and haematopoietic cells, with significantly lower levels of induction in epithelial cell types as well as in solid human cancers with an epithelial origin [47]. Our data shows that the radiosensitization induced by silencing cyclin D1 does not depend on apoptosis but rather by an increased accumulation of DNA-DSBs damage; RT treatment did not modify the percentage of apoptotic cells regardless of the expression of CCDN1, while it drastically increased DNA-DSBs damage, as suggested by the accumulation of activated H2AX protein [48]. Our data indicate that in PCa, such as in other cancers of epithelial origin, RT induces cell death by promoting DNA-DSBs phenomena; in this scenario cyclin D1 seems to be the guardian against RT-induced DNA damages. HR, through the ATM-RAD51-and NHEJ, via the DNA-PKcs-pathway, are the main highly conserved enzymatic pathways involved in repair of RT-induced DNA-DSBs [6–10]. We finally investigated the molecular partners of cyclin D1 responsible of cyclin D1-mediated radioresistance in PCa. A proteomic screen for cyclin D1 protein partners, performed in several types of human tumors, shows that cyclin D1 interacts with proteins implicated in DNA repair machinery such as RAD51 [17–22]. Herein, *in vitro* and *in vivo*, we show that cyclin D1 silencing abrogated the DNA-PKcs phosphorylation/activation (NHEJ pathway) and RAD51 accumulation (HR pathway) induced by RT. Although no modifications of ATM phosphorylation/activation status were showed, we found that cyclin D1physically interacts with posphorylated-activated-ATM as well as with activated-DNA-PKcs and RAD51: their associatedis increased in an RT dependent manner. Thus, cyclin D1 seems to play a dual role in controlling DNA-DSBs repair pathways; firstly by regulating NHEJ and HR pathways by sustaining the activation of DNA-PKcs, ATM and the expression basal levels of RAD51 and secondly by directly interacting with the DNA-DSBs repair machinery. The key role cyclin D1 in regulating androgen independent PCa cells radioresistance was also demonstrated by the observation that silencing of DNA-PKcs or RAD51 drastically increases the radiosensitivity of PCa cells. Thus, our evidence strongly suggests that cyclin D1 regulates the activity of downstream DDR machinery and promotes radioresistance of androgen independent PCa cells. The schematic showen in Figure 9 outlines the mechanism identified in our findings. Many issues should be further investigated such as the mechanisms by which cyclin D1 regulates the RT-induced ATM and DNA-PKCs-phosphorilation/activation, the mechanism by which cyclin D1 influences the RAD51 RT-induced DDR and the significance of the physical interaction between cyclin D1 and other members of DNA-DSBs repair machinery. Furthermore, cancer stem cell radioresistance has been described in several cancer types including prostate cancer [49–50] and it will be of interest to test whether silencing cyclin D1 in PCa cells that express stem-like properties improves radiosensitivity.

In the present study, we demonstrated that cyclin D1 governs the tumor phenotype and participates in determining radioresistance of PCa cells independently by androgen receptor expression. Our studies suggest silencing cyclin D1 may improve the therapeutic effects of RT.

## MATERIALS AND METHODS

### Cell culture and FACS analysis

The human prostate carcinoma cell lines 22Rv1, and PC3 were obtained from the American Type Culture Collection (Rockville, MD) and were grown in RPMI 1640 (Life Technologies, Inc., Grand Island, NY) supplemented with 10% FBS. For FACS analysis cells were harvested by trypsin-EDTA and washed; pellets were suspended in 0.3 ml 50% FCS in PBS, followed by addition of 0.9 ml 70% ethanol and left O/N in the dark at 4°C prior to FACS analysis (Coulter Epics XL Flow Cytometer, Beckman Coulter CA, USA).

### Viral production and infection and siRNA interference

Design of nucleotide sequence for cyclin D1short hairpin RNA (GCCACAGATGTGAAGTTCA) was performed with the BLOCK-iT™ RNAi Designer from Invitrogen. shRNA was designed that incorporated these sequences within a short hairpin structure, using the stem loop sequence 5′-TCAAGAGA-3′, which were then cloned between MluI and ClaI sites downstream of an H1 promoter in the plasmid pLVTHM. Plasmid pLVTHM, derived from pSUPERn that contains a GFP expression cassette upstream of the H1 promoter, was obtained from Addgene. The construct was stably transduced into PC3 and 22Rv1 cells using a lentiviral based expression system [26]. 293T cells were transfected using calcium phosphate transfection together with the packaging vectors psPAX2 (virus packaging plasmid) and pMD2G (Addegene) (envelope plasmid; 4:3:2 ratio) by calcium–phosphate transfection. Culture medium containing virus was collected 48 hours after transfection and filtrated through a 0.4 μm filter to remove cell debris and cells: viral titers were determined by measuring the percent of green fluorescent protein (GFP)–positive cells. Stable cell lines expressing shRNA were achieved by infection at 50 multiplicity of infection (MOI). The collected viruses were added to the target cells in the presence of polybrene (2 μg/mL) and incubated for 24 hours. Four days after the first infection, transduced cells were isolated by FACS sorting for GFP + cells to > 99% purity. RNA interference experiments were performed with siRNA for DNA-PKcs, ATM and RAD51 (Sancta Cruz Biotechnology) using Lipofectamine 2000 reagent (Invitrogen, Italy), according to the manufacturer's instructions. Briefly, cells were plated at 40–50% confluence and transfected after 24 hours with 100 nM siRNA, which we ascertained was sufficient to detect maximum fluorescence using fluorescein-conjugated control siRNA.

### Cell growth, soft agar colony formation, invasion and migration assays

Cells (20×10^4^/well) were plated in 24-well culture plates. The cell number was assayed by crystal violet staining at 2-day intervals up to 20 days. Medium was changed every 4 days. Control cells were analyzed under the same conditions until they reached confluence. In order to evaluate the ability of individual cell lines to grow in an anchorage-independent manner, cells were plated in soft agar. The bottom of each well contained 2 ml of 0.5% agarose and RPMI 2X (Sigma Aldrich Corporation, St Louis, MO, USA). This compact agar was covered with 2 ml of 0.2% agar and RPMI 2X with 5 × 10^3^ cells. The medium was changed every 3 days. After 21 days, the wells were stained with 0.003% crystal violet, and five areas were randomly selected from each well in order to count the approximate number of colonies. The cell-migration and invasion assays were performed using the CytoSelect™ ECM Cell Migration and Invasion Assay (8 μm, Colorimetric Format) (CELL BIOLABS, INC.) in accordance with manufactures instructions.

### Immunoblotting

Immunoblotting was performed as previously described [27] using the following antibodies: anti-cyclin D1(DCS-6), anti-ATM (H-248), anti-DNA-PKcs (E-6), anti-H2AX (M-20), anti-p-H2AX (3C10), anti-RAD51 (3C10), anti-Ku86 (B-1), anti-p21waf1 (B-2), anti-p27Kip1 (A-10) and α-tubulin (B-7) all from Santa Cruz Biotechnology. Anti-pospho-Ser1981-ATM (10H11.E12), anti-pospho-Thr2609-DNA-PKcs (10B1) were from abcam^®^. Peroxidase-conjugate anti-mouse or anti-rabbit IgG (Amersham-Pharmacia Biotech, UK or Santa Cruz) were used for enhanced chemiluminescence (ECL) detection.

### Immunoprecipitation

Cells were harvested in phosphate buffered saline, sedimented and lysed in 10 mM Tris pH 7, 50 mMNaCl, 1% NP40, 1 mM ZnCl_2_, in addition to protease and phosphatase inhibitors. Protein extracts were clarified by centrifugation. Supernatant, normalized as equal amounts of proteins, were incubated with antibody anti-cyclin D1(H-295RabbitPolyclonal) at 4°C for 3 hrs. 30 μl of protein-G Plus (Santa Cruz Biotechnology) were added to collect immunocomplexes. Protein G-bound immunocomplexes were washed 6 times with extraction buffer and processed for SDS-PAGE. Immunoblotting was performed with anti-RAD51 (3C10) from Santa Cruz Biotechnology or with anti-pospho-Ser1981-ATM (10H11.E12), anti-pospho-Thr2609-DNA-PKcs (10B1) from abcam^®^: all the antibodies used for the immunoblotting were mouse monoclonal.

### *In vitro* irradiation and colony formation assay

Radiation was delivered at room temperature using an x-6 MV photon linear accelerator, as previously described [28]. The total single dose of 4 Gy was delivered with a dose rate of 2 Gy/min using a source-to-surface distance (SSD) of 100 cm. A plate of Perspex thick 1.2 cm was positioned below the cell culture flasks in order to compensate for the build-up effect. Tumor cells were then irradiated placing the gantry angle at 180°. Non-irradiated controls were handled identically to the irradiated cells with the exception of the radiation exposure. For clonogenic survival assays, exponentially growing cells in 25-cm^2^ flasks were harvested by exposure to trypsin and counted. They were diluted serially to appropriate densities and plated in triplicate in 6 multi-well plates with 2 mL of complete medium/each well. After incubation for 24 hours, the cells were exposed at room temperature to radiation treatment as already described. The cells were then washed with PBS, cultured in growth medium for 14 days, fixed with methanol:acetic acid (10:1, v/v), and stained with crystal violet. Colonies containing > 50 cells were counted.

### Cell viability, apoptosis, caspases- and γ-H2AX activation assays

A Cell Proliferation Kit I (MTT) (Sigma-Aldrich) was used to quantitatively measure cellular viability. Tunnel assay (Promega) was used to quantitatively measure the cellular apoptosis. Caspase-Glo^®^3, 8 and 9 assays from Promega were used to measures caspase activity. All assays were performed accordingly to the manufacturer's instructions.

### Establishment of tumor xenografts and *in vivo* radiation treatment

PCa cells were grown to 80% confluence and harvested. Cells were re-suspended in serum free RPMI-1640 medium with penicillin and streptomycin, mixed 1:1 with Growth Factor Reduced (GFR) BD Matrigel Basement Membrane Matrix (BD Biosciences, Palo Alto, California). Using a cold syringe and 27-gauge needle, 3.5 × 10^6^ PC3 and 5 × 10^6^ 22Rv1 cells were injected subcutaneously into each lateral flank of male athymic nu/nu mice 6 weeks of age. Mice were kept under sterile conditions, receiving sterile nutrition and water. When palpable tumors (0, 5–1, 0 cm^3^) were established animals were subjected to radiation treatment. Mice were irradiated by two field (AP/PA) at room temperature using an Elekta6-MV photon linear accelerator. Five fractions of 2 Gy were delivered over 5 consecutive days for a total dose of 10 Gy with a dose rate of 1.5 Gy/min. Prior to irradiation, mice were anesthetized and were shielded from off-target radiation by a multi-leaf secondary collimator. Before tumor inoculation mice were randomly assigned to 2 experimental groups, with or without radiation treatment. Each group was composed of 10 mice. Experiments were stopped 20 days after the last RT fraction and mice were sacrificed by carbon dioxide inhalation. Tumours were directly frozen in liquid nitrogen for protein analysis and biochemical evaluation. All the procedures involving animals and their care were conducted in accordance with the institutional guidelines.

### Evaluation of treatment response *in vivo*

The effects on tumour growth of different treatments were evaluated as follows: (1) measurement of tumour volume during and at the end of experiments. Tumor volume was assessed by measurement every 4 days with a Vernier calliper (length × width). The volume of the tumor was expressed in mm^3^ according to the formula 4/3π r^3^; (2) measurement of tumor weight at the end of experiment; (3) Time to progression (TTP), defining tumor progression (TP) an increase of greater than 100% of tumor volume with respect to baseline.

### Histological and immunohistochemical analysis

Serial 3 μm sections were stained with Hematoxylin and Eosin (H&E) and Masson's Trichrome in order to evaluate morphological aspects. For immunohistochemical (IHC) analysis, sections were incubated for 40 minutes in methanol and 3% hydrogen peroxidase solution and then rinsed in PBS. Samples were incubated 10 minutes in buffered citrate 0.01 M, pH 6, twice and rinsed in PBS. Sections were then treated with BSA (5%) for 10 minutes and finally incubated overnight with specific antibodies against cyclin D1 (H-295), RAD51 (H-92), Ku86 (H-300), α-Actin (1A4) and p-ATM (10H11.E12) all from Santa Cruz Biotechnology Inc., Santa Cruz, CA, USA, γH2AX (IB 100–2280) from NOVUS biologicals, used at dilutions of 1:100. Samples were then rinsed with PBS for 5 minutes and incubated with a labeled streptavidin-biotin-peroxidase conjugate kit (Dako LSAB plus, cod.K0675, DakoCytomation, Milan, Italy). After rinsing in PBS for 10 minutes the sections were incubated with 3, 3-diaminobenzidine-tetrahydrochloride (DAB, Sigma Aldrich) for 1–3 minutes. The specificity of immune reactions was revealed by the absence of the primary antibodies. Lastly, the samples were counterstained with Mayer's Hematoxylin and observed under a photomicroscope Olympus BX51 Light Microscope (Olympus, Optical Co. Ltd, Tokyo, Japan). Observations were processed with an image analysis system (IAS, Delta system, Rome, Italy) and were independently performed by two pathologists (AV, RS) in a blinded fashion.

### *In vivo* densitrometric quantification of proteins

Densitometric analysis for α-SMA and cyclin D1 was obtained using “IHC Profile ImageJ” an automated digital program quantitates the intensity of antibody staining in tissue sections. The spectral deconvolution method of DAB/hematoxylinwas deployed, so that the DAB stained images could be separated and displayed independent of the hematoxylin image. Then the program selects where the marker protein is expressed the most, cytoplasm and/or nucleus. Therefore the deconvoluted image undergoes a pixel-by-pixel analysis, thus the full profile along with a scoring decision is provided. The results are shown in a four tier system which includes high positive, positive, low positive and negative.

### Colorimetric evaluation of collagen content

Samples (5 mm^3^) of tumor xenograft were removed and immediately immersed in 10% buffered formalin for paraffin embedding. Eight 15-mm thick, 100 mm^2^ large sections were obtained from each liver and used for colorimetric evaluation. Sections for colorimetric evaluation were deparaffinized through successive baths in absolute toluene: ethanol (50:50) and 50% aqueous ethanol and water. Staining procedures with fast green FCF 0.1 (Chroma- Gesellshaft, no. IA30, Stuttgart, Germany) and Sirius Red F3B 0.01% (Atomergic Chemical Corporation, no. 10022; Plainview, NY, USA) were performed according to Gascon-Barrè et al. [29]. Colors were eluted in 0.05 MNaOH and 50% aqueous methanol. The eluted colors were examined in Lambda 4 B PE spectrophotometer. Correlation between absorbance and protein estimations were assessed according to Gascon-Barrè et al. [29]. Non-collagenous protein determination was obtained using the following formula: Non-collagenous protein (mg) = Absorbance at 605 nm / 2.08. Collagenous protein determination was obtained using the latter interference factor in the following formula: Collagen (μg) = (Absorbance at 540 nm) – (0.26 absorbance at 605 nm) / 38.4 Collagen content (collagen /protein ratio) was calculated using the following formula: Collagen content (μg/mg total protein) = μg collagen / (μg collagen + mg non-collagenous protein) [29].

### Statistical methods

Continuous variables were summarized as mean and standard deviation (SD) or 95% CI for the mean. For continuous variables, statistical comparisons between control and treated groups were established by carrying out the ANOVA Tests (a parametric one-way analysis of variance for independent groups). Dichotomous variables were summarized by absolute and/or relative frequencies. For Dichotomous variables, statistical comparisons between control and treated groups were established by carrying out the exact Fisher's test. For multiple comparisons the level of significance was corrected by multiplying the *P* value by the number of comparisons performed (n) according to Bonferroni correction. TTP was analyzed by Kaplan-Meier curves and Gehan's generalized Wilcoxon test. When more than two survival curves were compared the Logrank test for trend was used. This tests the probability that there is a trend in survival scores across the groups. All tests were two-sided and were determined by Monte Carlo significance. *P* values < 0.05 were considered statistically significant.

For TTP, fractional TTP (FTTP) for each treatment group was calculated as the ratio between the median TTP of untreated and treated tumors. This was done for treatment a, for treatment b and for treatment a + b. The expected FTV or FTTP for the << a + b >> combination was defined as FTVa-observed X FTVb-observed or as FTTPa-observed X FTTP-observed. The ratio FTV a + b-expected/FTV a + b-observed or FTTP a + b-expected/FTTP a + b-observed was the combination Index (CI). If CI > 1, there are supra-additive effects and if CI < 1 infra-additive ones. Strictly additive effects are observed if CI = 1. All statistical analyses were performed using the SPSS^®^ statistical analysis software package, version 10.0.
