# Correction: Cyclin D1 silencing suppresses tumorigenicity, impairs DNA double strand break repair and thus radiosensitizes androgen-independent prostate cancer cells to DNA damage

**DOI:** 10.18632/oncotarget.12267

**Published:** 2016-09-27

**Authors:** Francesco Marampon, Giovanni Luca Gravina, Xiaoming Ju, Antonella Vetuschi, Roberta Sferra, Mathew C. Casimiro, Simona Pompili, Claudio Festuccia, Alessandro Colapietro, Eugenio Gaudio, Ernesto Di Cesare, Vincenzo Tombolini, Richard G. Pestell

Present: Due to an error made during submission of the revised manuscript, Dr. Giovanni Gravina's name was spelled incorrectly.

Correct: The correct author list for this manuscript is provided below. Authors sincerely apologize for this oversight.

Original article: Oncotarget. 2016; 7(5):5383-400. doi: 10.18632/oncotarget.6579.

Francesco Marampon^1,2,*^, Giovanni Luca Gravina^1,*^, Xiaoming Ju^2^, Antonella Vetuschi^1^, Roberta Sferra^1^, Mathew C. Casimiro^2^, Simona Pompili^1^, Claudio Festuccia^1^, Alessandro Colapietro^1^, Eugenio Gaudio^3^, Ernesto Di Cesare^1^, Vincenzo Tombolini^4^, Richard G. Pestell^2^

^1^ University of L'Aquila, Department of Biotechnological and Applied Clinical Sciences, L'Aquila, Italy

^2^ Department of Cancer Biology, Medical Oncology and Sidney Kimmel Cancer Center, Thomas Jefferson University, Philadelphia, Pennsylvania, USA

^3^ Department of Human Anatomy, “La Sapienza” University of Rome, Rome, Italy

^4^ Department of Radiotherapy, Policlinico Umberto I “Sapienza” University of Rome, Rome, Italy

^*^ These authors contributed equally to this work

